# Preparation and Performance Optimization of Fe^2+^:ZnSe Solid Solution by High-Pressure–High-Temperature Method

**DOI:** 10.3390/ma18040896

**Published:** 2025-02-19

**Authors:** Lijuan Wang, Haohao Yang, Shiyun Zheng, Xin Fan, Qiong Gao, Fangbiao Wang, Qi Chen, Peng Liu, Linjun Li

**Affiliations:** 1 Key Laboratory of Photonic and Electronic Bandgap Materials, Ministry of Education, School of Physics and Electronic Engineering, Harbin Normal University, Harbin 150025, China; 18645262755@163.com (L.W.); gaoqiong1988@163.com (Q.G.); 2 College of Physics and Electronic Engineering, Qujing Normal University, Qujing 655011, China; 15693840614@163.com (H.Y.); zsy979zsy@126.com (S.Z.); 3 Key Laboratory of New Carbon-Based Functional and Super-Hard Materials of Heilongjiang Province, School of Physics and Electronic Engineering, Mudanjiang Normal University, Mudanjiang 157011, China; fanxin_msy23@163.com (X.F.); wangfangbiao@126.com (F.W.); 4 Jiangsu Key Laboratory of Advanced Laser Materials and Devices, School of Physics and Electronic Engineering, Jiangsu Normal University, Xuzhou 221116, China

**Keywords:** zinc selenide, iron ion doping, concentration, structure, properties

## Abstract

In this paper, high-purity zinc selenide (ZnSe) prepared by the Chemical Vapor Deposition (CVD) method was used as the raw material, and iron ion-doped zinc selenide polycrystals were successfully fabricated through the thermal diffusion method at 1100 °C for 30 h. The results showed that iron ions (Fe^2+^) successfully penetrated into the zinc selenide crystals, but the concentration of iron ions inside the crystals was relatively low, and the crystals exhibited numerous defects. To address this issue, we performed secondary sintering and annealing on the samples under high-temperature and high-pressure (HPHT) conditions, with the annealing temperature range set at 900–1200 °C. The results demonstrated that, under the synergistic effects of high temperature and high pressure, the lattice spacing in the crystals significantly decreased, defects were reduced, the distribution of iron ions became more uniform, and the concentration of iron ions in the central region increased. Additionally, the density and hardness of the samples were significantly improved. The method of secondary sintering under high-temperature and high-pressure provides a novel approach for the preparation of iron ion-doped zinc selenide polycrystalline ceramics, contributing to the enhancement of ceramic properties.

## 1. Introduction

ZnSe, as a significant direct bandgap II-VI semiconductor material, exhibits a bandgap of 2.67 eV and an exciton binding energy of 21 meV at room temperature [1,2]. Its intrinsic emission in the blue or blue-green spectral region, coupled with excellent optical and electrical properties, makes it a promising candidate for luminescent materials, laser materials, and nonlinear optical applications [3,4,5,6,7]. ZnSe holds immense potential for both fundamental research and practical applications, including blue light-emitting devices, infrared thermal imagers, all-weather optical systems, short-wavelength lasers, and transmissive window materials [8,9,10,11,12,13,14]. In particular, Fe^2+^-doped ZnSe ceramics have garnered considerable attention due to their ability to enhance laser output efficiency and serve as gain media [15], making Fe^2+^:ZnSe a focal point of research for national security and economic development [16,17,18,19,20].

Traditional methods for preparing Fe^2+^:ZnSe crystals include physical vapor transport, melt growth, the Bridgman method, thermal diffusion, and hot-pressed ceramics [21,22]. Among these, the thermal diffusion method is the most mature technique for fabricating ZnSe polycrystalline materials. This method utilizes ZnSe single crystals or polycrystals as raw materials and involves long-term heat treatment in evacuated quartz tubes to achieve iron ion diffusion [23,24,25,26]. However, this approach suffers from limitations such as low impurity concentration, non-uniform diffusion, prolonged preparation periods, and size constraints [27,28,29]. To address these challenges, we propose a novel approach that combines thermal diffusion with high-pressure–high-temperature (HPHT) secondary annealing. HPHT technology has been widely employed to enhance the performance of ceramics, particularly by improving mechanical strength, modifying lattice spacing, and facilitating ion diffusion within the crystal lattice to optimize ion-doped materials.

Recent studies by Wang et al. [30] have demonstrated the potential of high-pressure–high-temperature (HPHT) annealing in optimizing the microstructure and properties of wide-bandgap semiconductors. Building on these advancements, in this study, Fe^2+^:ZnSe crystals were prepared using the thermal diffusion method, followed by HPHT annealing at 900–1200 °C and 2.0 GPa. This method not only overcomes the limitations of traditional thermal diffusion but also significantly improves doping uniformity and reduces defects. The results demonstrate that HPHT annealing promotes the uniform diffusion of iron ions, reduces lattice spacing, and enhances the optical and mechanical properties of ZnSe ceramics. Compared to conventional methods, HPHT offers a more efficient and scalable solution for optimizing Fe^2+^:ZnSe materials, providing a novel strategy for the preparation of high-performance II-VI semiconductors.

## 2. Materials and Methods

Thermal diffusion is a commonly used method for preparing doped semiconductor materials, and it can also be applied to fabricate iron-doped zinc selenide (Fe^2+^:ZnSe) laser crystals. The basic principle of this method involves the diffusion of ferrous ions from the high-temperature region to the low-temperature region of ZnSe crystals through thermal evaporation at elevated temperatures, thereby achieving doping. The preparation of ZnSe by thermal diffusion includes the following steps: First, the dopant powder containing ferrous ions and high-purity ZnSe crystals needs to be prepared. These materials must be treated under an inert atmosphere (e.g., argon) to prevent oxidation and other contamination. The dopant containing ferrous ions is placed in the high-temperature zone of the reactor, while the zinc selenide crystals are placed in the low-temperature zone. This temperature gradient is the key to the thermal diffusion process, driving the migration of dopant ions from the high-temperature zone to the low-temperature zone. At high temperatures, the ferrous ions in the dopant evaporate and diffuse into the zinc selenide crystals due to the temperature gradient. This process must be conducted under vacuum conditions to avoid the introduction of impurities. In this study, the diffusion temperature was 1100 °C, and the duration was 30 h. After thermal diffusion, the obtained iron-doped zinc selenide crystals were cut and assembled into composite blocks with a pressure transfer medium, then placed into a domestic six-anvil hydraulic (Guilin Guiye Machinery Co., Ltd., Guilin, China) press for the experiments. The constant pressure was set at 2.0 GPa, the temperature ranged from 900 to 1200 °C, and the experimental duration was 30 min.

**Figure 1:**
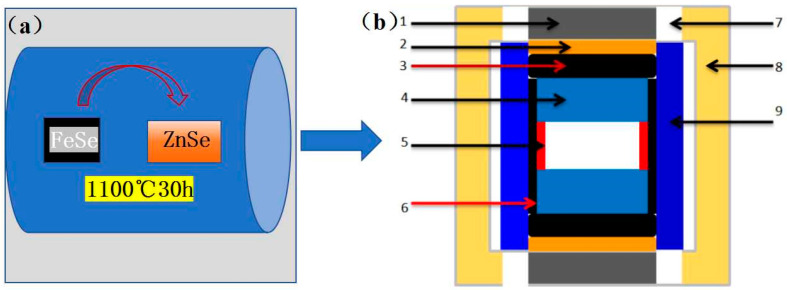
Flow chart of sample preparation process: (**a**) thermal diffusion preparation; process (**b**) 1—chlorite block; 2—graphite flake; 3—dolomite ring; 4—steel cap; 5—copper flake; 6—NaCl + ZrO_2_ lined tube; 7—graphite tube; 8—insulated tube; 9—sample.

The experimental samples were polished for X-ray diffraction (XRD) testing, energy-dispersive X-ray spectroscopy (EDS), Raman spectroscopy, scanning electron microscopy (SEM), and transmission electron microscopy (TEM). These techniques were used to analyze the structural composition, degree of crystallinity, microscopic morphology, and crystal type of the samples. The sample preparation process is illustrated in Figure 1.

## 3. Analysis and Discussion of Results

### 3.1. Comprehensive Characterization of ZnSe

#### 3.1.1. X-Ray Analysis

**Figure 2:**
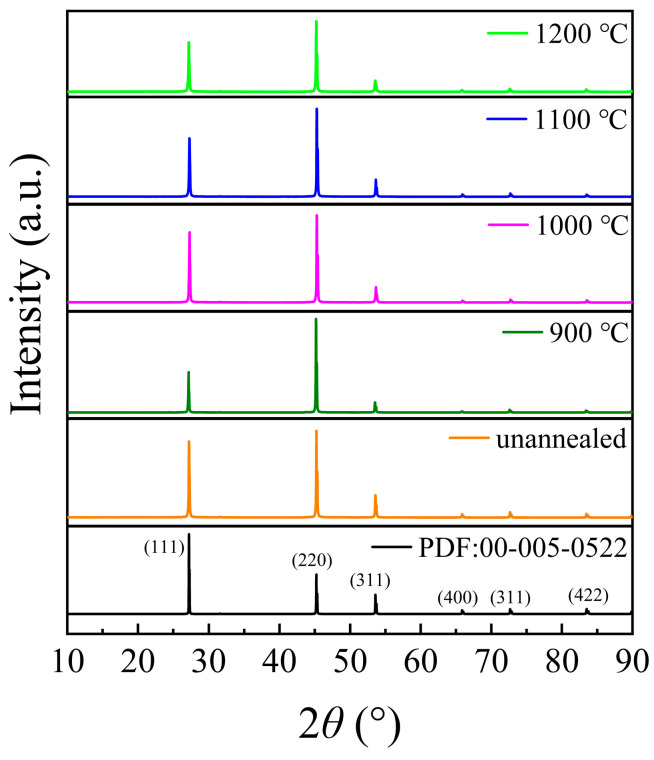
The XRD pattern of the sample (ZnSe).

The polished ZnSe sample was first subjected to X-ray diffraction (XRD) analysis to determine its composition and crystallinity, with a scanning angle range of 10–90°, as shown in Figure 2. Comparison with the standard diffraction patterns reveals distinct peaks at 27.20°, 45.17°, 53.53°, 65.82°, and 72.59°, which correspond to the (111), (220), (311), (400), and (331) planes of cubic sphalerite ZnSe, respectively. The absence of impurity peaks in the XRD pattern confirms that the crystal structure remains stable after annealing at 900–1200 °C, and no decomposition of ZnSe crystals occurs.

#### 3.1.2. Raman Spectroscopy Analysis

**Figure 3:**
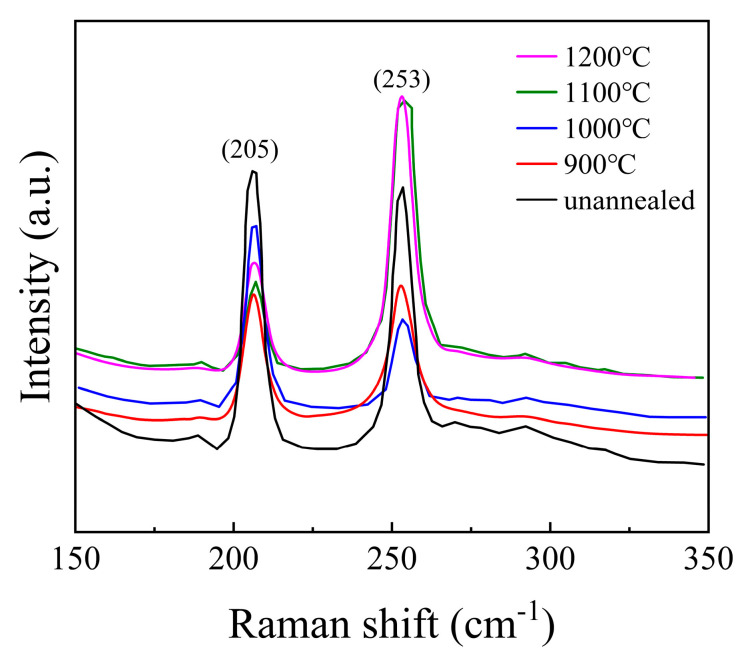
The Raman spectra of the samples (ZnSe).

**Figure 4:**
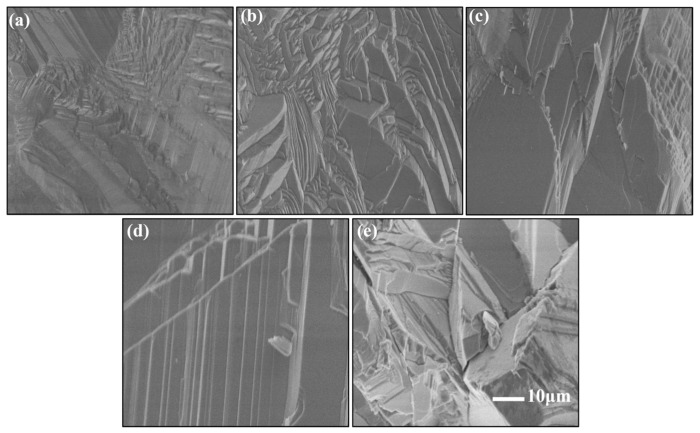
Surface morphology (SEM) of ZnSe samples after different annealing temperatures: (**a**) unannealed, (**b**) 900 °C, (**c**) 1000 °C, (**d**) 1100 °C, and (**e**) 1200 °C.

To investigate the structural characteristics of the samples, Raman spectroscopy was performed, and the results are presented in Figure 3. For ZnSe crystals with a zinc blende (sphalerite) structure, the Raman peaks located at 205 cm^−1^ and 253 cm^−1^ correspond to the transverse optical (TO) and longitudinal optical (LO) phonon vibrational modes, respectively. Similar observations were reported by Shakir et al. [31,32], which confirms the consistency of our findings. The absence of vibration peaks related to impurities indicates that the structure of the ZnSe crystals is relatively stable and free from significant contamination. At temperatures of 900 °C, 1000 °C, and 1100 °C, the positions of the vibrational peaks of the ZnSe crystals remain nearly unchanged. However, the full width at half maximum (FWHM) of the peak at 205 cm^−1^ decreases with increasing temperature, while the FWHM of the peak at 253 cm^−1^ increases. This behavior can be attributed to the differences in the vibrational characteristics of the TO and LO phonon modes. Specifically, the TO mode is more sensitive to structural perfection, and its narrowing FWHM suggests a reduction in defect density or an improvement in crystalline quality at higher temperatures. In contrast, the broadening of the LO mode may indicate increased phonon scattering due to thermal effects or the presence of residual stress. When the temperature reaches 1200 °C, a slight red shift is observed in the Raman peak at 253 cm^−1^. This red shift is likely caused by overheating of the ZnSe crystals, leading to an increase in internal defects or structural changes that affect the LO phonon mode. These findings are consistent with the results described in Figure 4, further supporting the correlation between temperature and structural integrity in ZnSe crystals.

#### 3.1.3. Microscopic Morphology Analysis

To investigate the microstructural evolution of the samples under different annealing temperatures, scanning electron microscopy (SEM) was employed for characterization, as shown in Figure 3. In the unannealed sample, ZnSe grains exhibit a mixed morphology of large and small grains: small columnar crystals with dimensions of approximately 4–5 μm are surrounded by similarly sized small columnar crystals, while larger columnar crystals of 30–50 μm are distributed at the periphery. The surfaces of the small columnar crystals display irregular polygonal shapes with high morphological similarity, whereas the large grains exhibit irregular shapes with significant variations. Although the grains are closely connected, voids are still present between them. At an annealing temperature of 900 °C, the sample morphology resembles that of the unannealed sample, with non-uniform grain size distribution and a small number of voids within the crystals. The polycrystalline grains exhibit synchronous growth characteristics, where adjacent grains merge and grow under the influence of sintering forces. However, as the spatial distance between grains decreases, further grain fusion does not occur, and grain boundaries remain clearly visible, indicating an insufficient driving force for further grain coalescence. This demonstrates the critical role of temperature in the fabrication of high-quality ZnSe ceramics. When the annealing temperature is increased to 1000 °C, partial fusion of small grains into larger grains is observed, accompanied by a reduction in grain gaps and smoother surfaces of some crystals, leading to improved densification. At an annealing temperature of 1100 °C, the grain size increases significantly, with columnar grains showing uniform distribution and dimensions of approximately 10–20 μm. The grain boundaries are distinct, and the density is exceptionally high, indicating optimal sintering conditions. However, at an annealing temperature of 1200 °C, the sample morphology is characterized by the interphase distribution of large grains, increased grain gaps, higher void density, and elevated defect concentrations. This is likely due to abnormal grain growth caused by excessive calcination temperatures, resulting in over-sintering and the subsequent degradation of crystal properties.

### 3.2. Distribution of Iron Ions in ZnSe Crystals and Influence of Annealing: EDS and Transmission Electron Microscopy Studies

#### 3.2.1. EDS Analysis of Iron Ion Distribution

**Figure 5:**
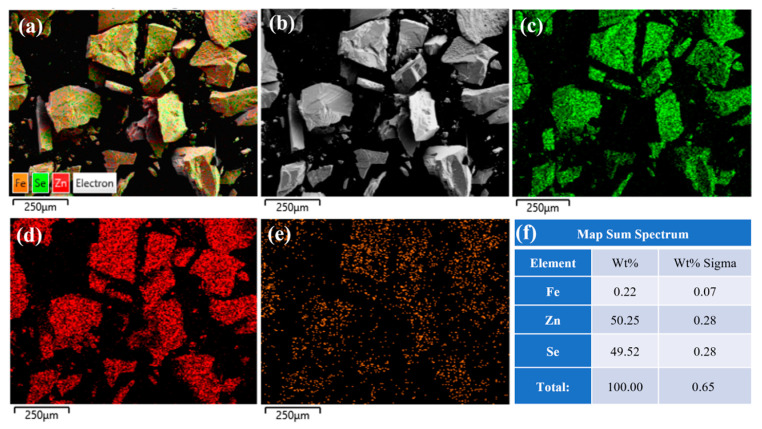
The EDS spectrum of the surface of the unannealed ZnSe sample; (**a**) Fe^2+^: ZnSe full element distribution map; (**b**) Surface morphology of Fe^2+^:ZnSe; (**c**) Distribution map of Se element; (**d**) Distribution map of Zn element; (**e**) Distribution map of Fe element; (**f**) Identification table of EDS elemental composition for selected areas.

To investigate the distribution characteristics of iron ions within the crystals, energy-dispersive X-ray spectroscopy (EDS) was employed to analyze Fe^2+^:ZnSe crystals prepared by the diffusion method. The EDS spectra of the surface of the unannealed sample are shown in Figure 5. Figure 5a–e reveals that iron (Fe), zinc (Zn), and selenium (Se) elements are uniformly distributed on the crystal surface. As indicated in Figure 5f, the mass fraction and atomic fraction of iron ions are 0.22% and 0.07%, respectively, confirming a relatively high concentration of iron ions on the crystal surface. This is primarily attributed to the diffusion of iron ions from the surface to the interior under high-temperature conditions.

**Figure 6:**
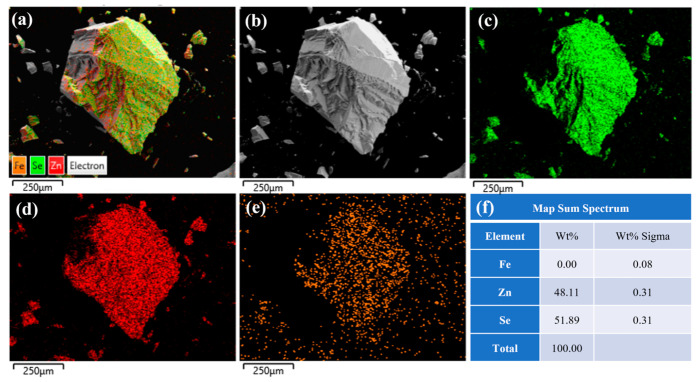
The EDS spectrum of the center of the unannealed ZnSe sample; (**a**) Fe^2+^: ZnSe full element distribution map; (**b**) Surface morphology of Fe^2+^:ZnSe; (**c**) Distribution map of Se element; (**d**) Distribution map of Zn element; (**e**) Distribution map of Fe element; (**f**) Identification table of EDS elemental composition for selected areas.

Figure 6 presents the EDS spectral analysis results at the center of the same sample. The figure reveals a uniform distribution of iron (Fe), zinc (Zn), and selenium (Se) elements on the crystal surface. As shown in Figure 6f, the mass fraction of iron ions is 0, while the atomic fraction is 0.08%, indicating slow diffusion and a low concentration of iron ions at the center of the ZnSe crystals prepared by the diffusion method. A comparison between Figure 5 and Figure 6 demonstrates that the Fe^2+^:ZnSe crystals prepared by the thermal diffusion method exhibit a higher iron ion concentration on the surface and a lower concentration at the center. This is attributed to the relatively small temperature driving force of the thermal diffusion method (T = 1100 °C), which facilitates the penetration of iron ions into the sample surface. However, the central region, being farther from the surface, experiences greater ion diffusion resistance, resulting in a gradual decrease in the iron ion concentration from the surface to the center and non-uniform distribution.

#### 3.2.2. EDS Analysis of Ferric Ion Distribution After Secondary Annealing at High Temperature and Pressure

**Figure 7:**
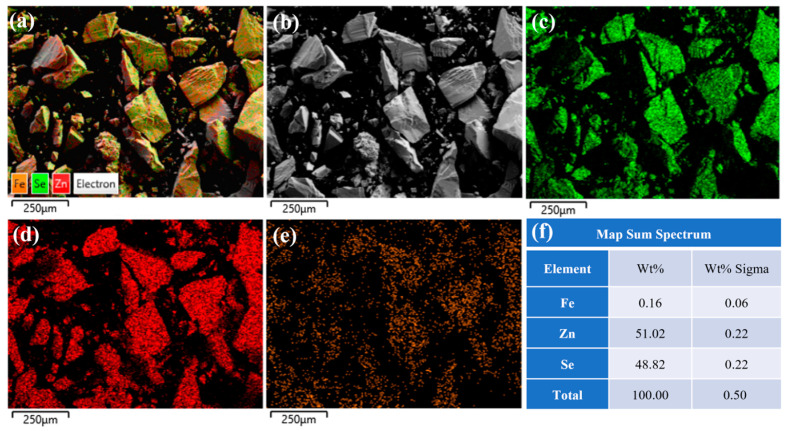
The EDS spectrum of the center of the ZnSe sample after annealing at 1100 °C; (**a**) Fe^2+^: ZnSe full element distribution map; (**b**) Surface morphology of Fe^2+^:ZnSe; (**c**) Distribution map of Se element; (**d**) Distribution map of Zn element; (**e**) Distribution map of Fe element; (**f**) Identification table of EDS elemental composition for selected areas.

Figure 7 presents the EDS spectrum of the crystal center under high-temperature and high-pressure secondary annealing conditions at 1100 °C. As shown in Figure 7f, the mass fraction and atomic fraction of iron ions are 0.16% and 0.06%, respectively, with a uniform distribution. A comparison with Figure 6 reveals an increase in iron ion concentration at the sample center. This phenomenon is attributed to the reduction in lattice spacing and crystal defects during the high-temperature and high-pressure secondary annealing process, which promotes the diffusion of iron ions from the surface to the center under the combined effects of high temperature and pressure gradients, ultimately achieving uniform distribution of iron ions within the crystal. Therefore, high-temperature and high-pressure secondary annealing effectively improves the ion concentration distribution in Fe^2+^:ZnSe crystals and enhances crystal quality.

#### 3.2.3. Transmission Electron Microscopy Analysis of ZnSe Crystals Before and After Annealing

**Figure 8:**
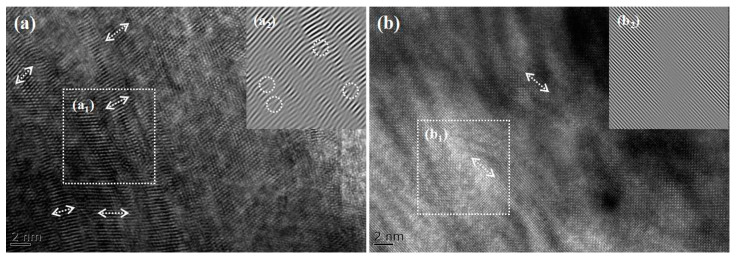
TEM patterns of samples (ZnSe): (**a**) Fe^2+^: ZnSe prepared by thermal diffusion method; (**a_1_**) is a partial image of (**a**), and (**a_2_**) is the Fourier transform of (**a_1_**); (**b**) Fe^2+^: ZnSe annealed at 1100 °C by high-temperature and high-pressure method; (**b_1_**) is a partial image of (**b**), and (**b_2_**) is the Fourier transform of (**b_1_**).

Figure 8 presents the transmission electron microscopy (TEM) images of the samples, where Figure 8a shows the unannealed crystals and Figure 8b displays the crystals annealed at 1100 °C. Figure 8(a2,b2) shows the Fourier transform results of the selected regions, respectively. A comparison between Figure 8a,b reveals that the crystals exhibit a polycrystalline structure, as is consistent with the XRD test results. Figure 8(a_1_) indicates irregular lattice arrangements with severe distortions and high distortion concentrations, which may arise from two factors: first, the high temperature and prolonged processing required for crystal preparation via the thermal diffusion method increase internal defects; second, the non-uniform distribution of iron ion concentrations within the crystals leads to lattice distortions. Figure 8(b_2_) demonstrates that the annealed ZnSe crystals exhibit more ordered lattice arrangements, reduced distortion concentrations, and smaller lattice spacings. These results indicate that high-temperature and high-pressure annealing effectively reduces lattice spacing and internal defects, promoting uniform diffusion of iron ion concentrations and thereby enhancing crystal properties.

### 3.3. ZnSe Polycrystalline Ceramics: Hardness, Density, and Densification

Table 1 presents the hardness, density, and densification properties of Fe^2+^:ZnSe polycrystalline ceramics. The results indicate that after high-temperature and high-pressure annealing, the hardness, density, and densification of the crystals are significantly improved, with the hardness showing the most notable change. The reduction in lattice spacing, increase in bonding energy, and enhanced intergranular interactions under high-temperature and high-pressure conditions are the primary reasons for the improvement in hardness and density, which is consistent with the results shown in Figure 8. Under a pressure of 2.0 GPa, the hardness, density, and densification of the crystals initially increase and then decrease with rising annealing temperatures. The insufficient calcination temperatures of 900 °C and 1000 °C result in relatively poor densification, necessitating higher annealing temperatures. The optimal performance is achieved at 1100 °C, where the hardness reaches 210 HV, matching the theoretical hardness value of the crystal, and the density reaches 5.541 g/cm³. However, when the annealing temperature is increased to 1200 °C, abnormal grain growth and increased surface defects occur, weakening the temperature-driven diffusion effect on ZnSe crystallization. Consequently, further densification becomes challenging, and both density and hardness decrease, as is consistent with the results shown in Figure 4.

## 4. Conclusions

In this study, we proposed a novel approach combining thermal diffusion with high-temperature and high-pressure (HTHP) secondary annealing to fabricate iron ion-doped zinc selenide (Fe^2+^:ZnSe) ceramics using high-purity ZnSe polycrystals as raw materials. Initially, the samples were treated at 1100 °C for 30 h using the thermal diffusion method. The results revealed that the iron ion concentration in the ZnSe crystals prepared by the conventional diffusion method was non-uniform, with higher concentrations on the surface and lower concentrations in the center. To address this issue, we innovatively introduced HTHP secondary annealing, treating the samples under conditions of 900–1200 °C and 2 GPa. The findings demonstrate that this method significantly reduces internal defects, decreases lattice spacing, and promotes the uniform diffusion of iron ions within the crystals, thereby increasing the central Fe^2+^ concentration and improving crystal quality. This approach not only provides a new strategy for the preparation of Fe^2+^:ZnSe ceramics but also offers a scalable and cost-effective solution for optimizing other transition metal-doped II-VI semiconductors.

## Figures and Tables

**Table 1:** Physical properties of samples (ZnSe).

	Unannealed	900 °C	1000 °C	1100 °C	1200 °C
Hardness	137	160	170	210	190
Density(g/cm^3^)	5.32	5.46	5.50	5.54	5.49
Density (%)	99.1	99.2	99.4	99.7	99.4
Error Range (%)	±2.0	±1.5	±1.0	±0.8	±0.5

## Data Availability

The original contributions presented in this study are included in the article. Further inquiries can be directed to the corresponding authors.
